# Delayed Occurrence of Hypertrophic Olivary Degeneration after Therapy of Posterior Fossa Tumors: A Single Institution Retrospective Analysis

**DOI:** 10.3390/jcm8122222

**Published:** 2019-12-16

**Authors:** Martin A. Schaller-Paule, Christian Foerch, Sara Kluge, Peter Baumgarten, Jürgen Konczalla, Joachim P. Steinbach, Marlies Wagner, Anna-Luisa Luger

**Affiliations:** 1 Department of Neurology, University Hospital Frankfurt, Goethe-University, 60528 Frankfurt am Main, Germany; 2 Institute of Neuroradiology, University Hospital Frankfurt, Goethe-University, 60528 Frankfurt am Main, Germany; sara.kammerer@gmail.com (S.K.);; 3 Department of Neurosurgery, University Hospital Frankfurt, Goethe-University, 60528 Frankfurt am Main, Germany; peter.baumgarten2@kgu.de (P.B.); juergen.konczalla@kgu.de (J.K.); 4 Dr. Senckenberg Institute of Neurooncology, University Hospital Frankfurt, Goethe-University, 60528 Frankfurt am Main, Germany; joachim.steinbach@kgu.de (J.P.S.); anna-luisa.luger@kgu.de (A.-L.L.); 5 University Cancer Center Frankfurt (UCT), University Hospital Frankfurt, Goethe-University, 60528 Frankfurt am Main, Germany; 6 German Cancer Consortium (DKTK), Partner Site Frankfurt/Mainz, 60528 Frankfurt am Main, Germany; 7 Frankfurt Cancer Institute (FCI), University Hospital Frankfurt, Goethe-University, 60596 Frankfurt am Main, Germany

**Keywords:** palatal tremor, posterior fossa masses, Holmes tremor, medulloblastoma resection, HOD

## Abstract

(1) Background: A lesion within the dentato-rubro-olivary pathway (DROP) in the posterior fossa can cause secondary neurodegeneration of the inferior olivary nucleus: so-called hypertrophic olivary degeneration (HOD). The clinical syndrome of HOD occurs slowly over months and may be overlooked in progressive neuro-oncological diseases. Posterior fossa tumors are often located near these strategic structures. The goal of this study was to analyze the systematics of HOD occurrence in neuro-oncological patients. (2) Methods: The neuroradiological database of the university healthcare center was scanned for HOD-related terms from 2010 to 2019. After excluding patients with other causes of HOD, 12 datasets from neuro-oncological patients were analyzed under predetermined criteria. (3) Results: Patients received multimodal tumor treatments including neurosurgery, radiotherapy, and chemotherapy. HOD occurred both unilaterally (left *n* = 4; right *n* = 5) and bilaterally (*n* = 3). Though the mass effect of posterior fossa tumors had already affected strategic structures of the DROP, none of the patients showed signs of HOD on MRI until therapeutic measures including neurosurgery affecting the DROP were applied. HOD was visible on MRI within a median of 6 months after the neurosurgical intervention. In 67%, the presumed underlying surgical lesion in the DROP lay in the contralateral dentate nucleus. (4) Conclusion: In a selected cohort of neuro-oncological patients, therapeutic lesions within the DROP were associated with HOD occurrence.

## 1. Introduction

A lesion within the functional brainstem loop of the dentato-rubro-olivary pathway (DROP) leads to secondary trans-synaptic neurodegeneration of the inferior olivary nucleus, a condition called “hypertrophic olivary degeneration” (HOD) [1,2,3,4,5]. The DROP, or so-called Guillain–Mollaret triangle (GMT), consists of three anatomic structures: the medullar inferior olivary nucleus (ION), the mesencephalic red nucleus, and the contralateral dentate nucleus of the cerebellum [5,6,7,8]. Even though the underlying pathology is not yet completely understood, it is agreed that disinhibition of the ION leads to its degenerative hypertrophy and a change in the modulation of cerebellar functioning [1,9,10]. Analogously to the pathophysiology and directed course of the functional dentate-rubro-olivary-pathway, HOD occurs contralateral to the lesion of the dentate nucleus and ipsilateral of injury to the central tegmental tract in the pons [4]. HOD frequently occurs bilaterally and it is not uncommon that the causative lesion cannot be identified [11].

The main symptoms are a palatal tremor, a primarily vertical pendular nystagmus, and a Holmes (dentate–rubral) tremor of the upper limbs [12,13]. In cerebral MRI, hyperintensity of the ION can usually be observed as focal hyperintensity on T2-weighted images within the first month after the index event, followed by hypertrophy of the ION usually within first 6 months and atrophy or complete restitution after 3–4 years [7]. In a recent analysis of 12 patients with HOD after stroke, all patients showed radiological signs of HOD following a lesion to the GMT, but only in half of them were the clinical syndrome and HOD diagnosis recognized by the treating physician [14]. 

HOD may also develop in patients with brain tumors in the posterior fossa affecting the GMT, either primarily due to tumor infiltration of the DROP or secondarily as a result of therapeutic measures such as neurosurgery or radiation therapy [15]. 

HOD symptoms and imaging aspects could be misinterpreted as a result of tumor recurrence or even as a therapeutic side-effect. Because the signs of HOD are often subtle and might therefore be underrecognized especially if patients are asymptomatic, we retrospectively analyzed neuro-oncological patients with posterior fossa tumors and HOD to describe the underlying patterns of injury, treatment regimens, and the presented symptoms as well as pitfalls in interpreting clinical and radiological HOD aspects. 

## 2. Materials and Methods

### 2.1. Study Population 

**Figure 1:**
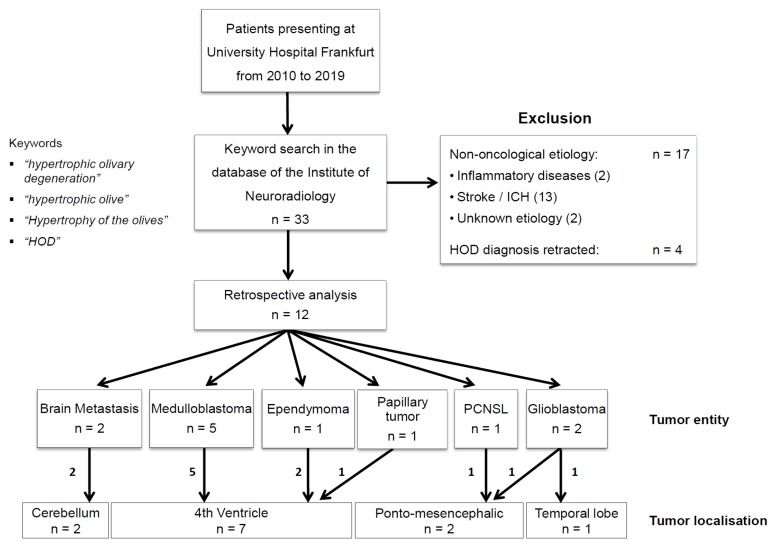
Patient selection. Patient selection of the current study is shown: The neuroradiological database of our university health care center was scanned for the terms “hypertrophic olivary degeneration”, “hypertrophic olive”, “hypertrophy of the olives”, and “HOD” from the years 2010 to 2019. In all, 33 cases were identified. Excluded were patients with stroke (*n* = 13), patients with inflammatory disease (*n* = 2), and patients with diseases of unknown etiology (*n* = 2). In four patients, the radiological diagnosis of HOD was retracted retrospectively. The remaining 12 patients had a radiological diagnosis of HOD and a cerebral tumor as the basic disease and were analyzed.

The neuro-radiological database of our university healthcare center was scanned for the terms “hypertrophic olivary degeneration”, “hypertrophic olive”, “hypertrophy of the olives”, and “HOD” from the years 2010 to September 2019. A total of 33 cases were identified. In all, 13 patients with stroke, 2 patients with inflammatory diseases, and 2 patients with diseases of unknown etiology were excluded. In four patients, the radiological diagnosis of HOD was retracted retrospectively. The remaining 12 patients had a confirmed radiological diagnosis of HOD and brain tumor. These 12 data sets were then collected and analyzed under predetermined criteria (Figure 1). The retrospective study was approved by the institutional Review Board of the Ethical Committee at the University Hospital Frankfurt (project number: 73/18).

### 2.2. Magnetic Resonance Imaging

Radiological diagnoses of HOD, including locating the lesions as well as the underlying patterns of injury, were analyzed by an experienced neuroradiologist (M.W.) and an experienced neurologist (M.S.-P.). HOD was diagnosed if a typical circumscribed hyperintensity of the inferior olivary nucleus in T2-weighted images with or without hypertrophy and without enhancement or diffusion restriction was present [6,7,11,15,16].

## 3. Results

### 3.1. Patient Characteristics 

Median age in our study was 38 years, and 33 percent of patients were female (Table 1). The underlying tumors included medulloblastoma (*n* = 5), glioblastoma (*n* = 2), brain metastasis (*n* = 2), primary CNS lymphoma (PCNSL; *n* = 1), papillary tumor of the pineal region (*n* = 1), and ependymoma (*n* = 1). All tumors were located in the posterior fossa (Figure 1, Table 1). Patients had received multimodal treatments in most cases, including neurosurgery, radiotherapy (RT), and chemotherapy (CH) before HOD was diagnosed (Table 1). 

### 3.2. Patterns of Lesion

**Figure 2:**
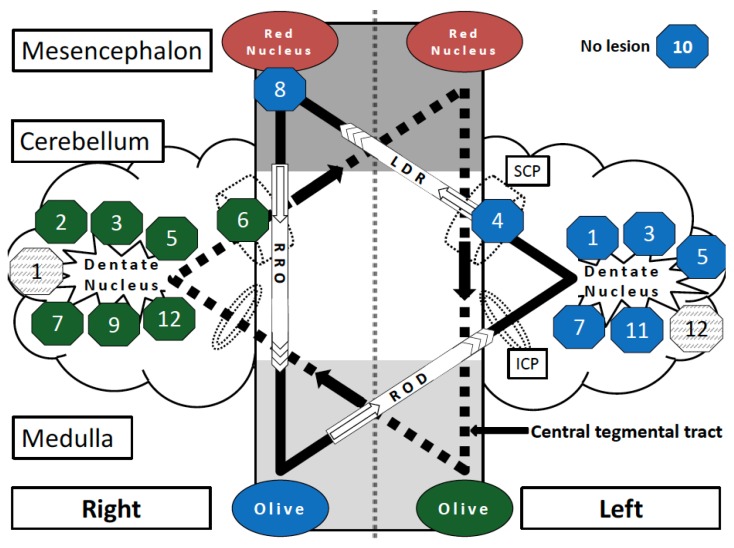
Schematic overview of the strategic lesions (not the tumors) in the Guillain–Mollaret triangle leading to HOD in the cohort of neuro-oncological patients. The bilateral overlapping Guillain–Mollaret triangles (dentate-rubro-olivary pathway) form a “star of David configuration”. The framed numbers describe the position of the HOD causing lesions in Patients 1–12. The background color of the octagons (green or blue) reflects the resulting degeneration of the left or right olive. Lesions that did not cause HOD are represented by grey shaded octagons. Codings are named according to pathway and side of origin: RRO = right rubro-olivary pathway (tractus rubroolivaris); ROD = right olivo-dentate pathway (tractus olivocerebellaris); LDR = left dentate-rubro pathway (fibrae cerebellorubrales); SCP = superior cerebellar peduncle; ICP = inferior cerebellar peduncle; olive = inferior olivary nucleus.

In five patients, HOD affected the right ION, in four patients the left ION, and in three patients, HOD was diagnosed bilaterally. In 67% of HOD cases (8 of 12), the underlying lesion within the dentato-rubro-olivary-pathway affected the dentate nucleus, in three of those patients it was one-sided, and in five cases it was bilateral. Two patients showed lesions in the cerebellar peduncle. In one patient, the red nucleus and central tegmental tract were affected; one patient did not show any focal lesions on MRI (MR-negative) (Table 2, Figure 2). Surgical approaches included transvermal (*n* = 3), telovelar (*n* = 4), paramedian (*n* = 1), and temporal (*n* = 1) approaches, and frontal/parietal biopsy (*n* = 2) (Table 1). 

### 3.3. Clinical Presentation

Leading symptoms of HOD occurred in 33% of cases (palatal tremor and Holmes tremor). The clinical diagnosis of HOD was discussed by the treating physicians in the discharge summary in 33% (4 out of 12 patients) of all cases. In 75% (3 out of 4) of these cases, patients showed leading symptoms of HOD (Table 2). 

### 3.4. Delay in Diagnosing HOD

The median latency between tumor diagnosis and retrospective radiological diagnosis of HOD was 15 months. The median latency of therapeutic (neurosurgical) intervention to first signs of HOD on MRI in retrospective was 6 months (Table 2). The median difference between first written diagnosis of HOD in radiological reports and retrospective imaging findings of HOD on prior MRIs was 6 months. 

### 3.5. Model of HOD Prediction

Based on the imaging findings, of all 12 cases a model allowing the side of HOD to be predicted according to individual lesion location was designed (Figure 2). The bilateral Guillain–Mollaret triangle can be illustrated as two overlapping triangles in a “star of David configuration” [14]. It is noteworthy that the original tumor location is not visualized, but only the localization of the destructive lesion through therapeutic (e.g., neurosurgical) intervention. The model schematizes that a single strategic lesion located at the crossroad between the two superior cerebellar peduncles can induce bilateral HOD by affecting both GMT [17,18].

### 3.6. Image Series before and after HOD Development 

**Figure 3:**
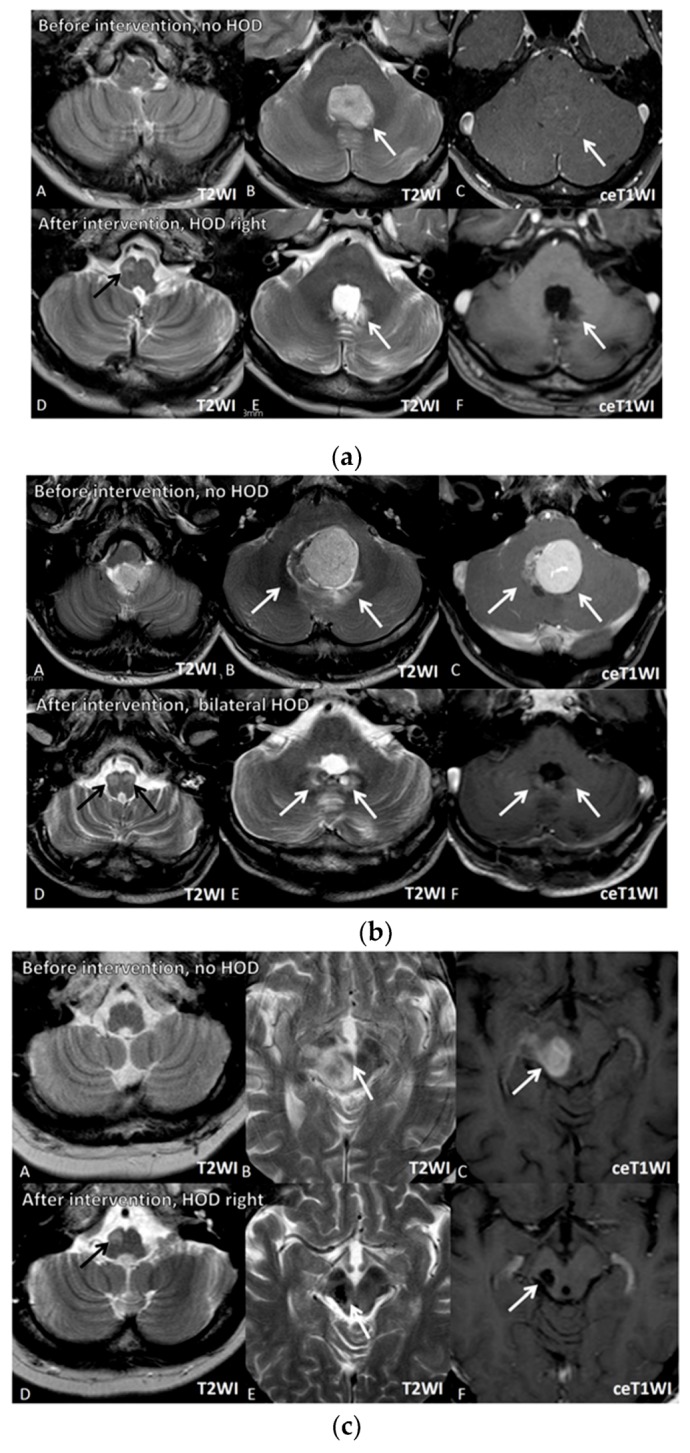
Image series of three illustrative case examples from the series. Image Series 1 represents Patient 11, Image Series 2 represents Patient 7, and Image Series 3 represents Patient 8 in Table 1. (**a**) Image Series 1: Upper row (07.09.2017): T2-(A,B) and contrast-enhanced (ce) T1-weighted images (WI) (C) of a 41 year old male patient (Table 1, patient 11) with non-enhancing medulloblastoma located in the fourth ventricle arising from the area of the left dentate nucleus (white arrows). At the time of tumor diagnosis, no signs of HOD were visible (A). Lower row (29.10.2018): After complete tumor resection and radiochemotherapy, small destructive defects (white arrows) were found in the left more than in the right dentate nuclei (E (T2WI), F (ceT1WI)). D (T2WI) reveals signs of right-sided HOD with focal T2 hyperintensity 3 months after resection (black arrow). (**b**) Image Series 2: Upper row (18.06.2012): T2-(A,B) and contrast-enhanced (ce) T1-weighted images (WI) (C) of a 35 year old male patient (Table 1, patient 7) with a medulloblastoma located in the fourth ventricle arising from the area of the left more than right dentate nuclei (white arrows). At this time, no signs of HOD were visible (A). Lower row (30.04.2013): After complete tumor resection and radiochemotherapy, small defects (white arrows) can be found in the left more than in the right dentate nuclei (E (T2WI), F (ceT1WI)). D (T2WI) reveals signs of bilateral HOD with focal swelling and T2 hyperintensities (black arrows). (**c**) Image Series 3: row (11.03.2013): T2-(A,B) and contrast-enhanced (ce) T1-weighted images (WI) (C) of a 56 year old patient (Table 1 patient 8) with primary lymphoma of the central nervous system located in the mesencephalon involving the right red nucleus (white arrows). At this time, no signs of HOD are visible (A). Lower row (30.08.2013): After biopsy and chemotherapy with complete response, a small post-traumatic hemorrhagic defect and hemosiderin (white arrows) including the right red nucleus and right central tegmental tract as well as atrophy of the right mesencephalic peduncle were detected (E (T2WI), F (ceT1WI)). D (T2WI) reveals signs of right-sided HOD with focal swelling and T2 hyperintensity one month after interventional therapy (black arrow).

In all 12 cases, there was no evidence of HOD on the MRIs at the time of tumor diagnosis. In all patients who received neurosurgical treatment, there was no HOD before the surgical treatment, which was documented in all patients by pre-operative planning MRI acquired shortly before the surgery (median 4 days) and post-operative control MRI without any signs of HOD (Table 2). However, HOD occurred within a median of 6 months after the therapeutic intervention affecting the Guillain–Mollaret triangle. Figure 3 exemplarily illustrates three patients developing HOD after therapeutic intervention, including neurosurgery and radiotherapy affecting the GMT. In all three patients, the GMT seem to have already been involved by tumor location or surrounding edema at time of radiological tumor diagnosis, but there were no signs of HOD (upper rows). Follow-up MRI in these cases showed occurrence of HOD within 1, 3, and 10 months after neurosurgery and radiotherapy with lesions of the dentate nuclei. The surgical approach, presumably affecting the dentate nuclei, is clearly visible on T2WI.

## 4. Discussion

Our study demonstrated that a HOD-specific symptom complex can occur from tissue damage within the functional loop of the DROP. The novelty of this analysis lies within the finding that HOD was not apparent when posterior fossa tumors affected the DROP, but then occurred within the expected time window as soon as multimodal therapeutic regimens were applied. 

Even though the entity of HOD was described over a century ago by the German physiologist Oppenheim in 1887 and confirmed by multiple authors in 1902 and 1903 [3,8,19], neither imaging findings nor the clinical syndrome are sufficiently recognized by physicians. In neuro-oncological patients, the development of novel neurological symptoms after treatment of posterior fossa masses is usually associated with tumor relapse. The clinical syndrome of HOD is characteristic but its identification requires a precise examination and awareness of possible HOD. Palatal tremor, Holmes tremor, and pendular nystagmus should be widely known and be associated with HOD, which requires an increased alertness to these specific symptoms as well as an understanding of the underlying pathophysiology. In our case series, only 4 out of 12 HOD patients were labeled with the diagnosis HOD on discharge, 3 of whom suffered from HOD associated symptoms. The MR finding of hyperintensity within the medulla without contrast enhancement after therapeutic intervention of posterior fossa masses should always be associated with HOD before thinking of tumor relapse or metastasis. Awareness of the entity is essential to avoid unnecessary diagnostic and therapeutic interventions. Additionally, the perception of neuroradiologists towards HOD can be improved, as our case series confirmed that first signs of HOD were retrospectively apparent on MRIs in median a full 6 months before the first explicit mentioning in a radiological report. 

Patients in this case series showed mainly lesions of the dentate nuclei, which led to contralateral HOD. Other lesions of the DROP were rarely found in our collective. Figure 2 illustrates that all but one case of HOD followed the causal hypothesis of a HOD causing a lesion within the dentato-rubro-olivary-pathway and its unidirectional orientation. Conclusively, no HOD was found in the literature with damage to the efferent olivocerebellar fibers, which do not functionally affect the olive itself [10,15]. One case was MR-negative, which could be explained either by a small radiotherapeutic lesion below the 3 Tesla detection threshold or between MRI slices, or the presence of idiopathic, non-traumatic HOD. It is not uncommon that a specific DROP lesion leading to HOD is not evident; in a retrospective review of 102 patients with HOD by Carr et al., a lesion could not be identified in 44% of cases [11]. It is noteworthy that if a lesion could be identified, HOD was due to a variety of etiologies in this study, such as trauma/surgery (32%), vascular malformation (21%), and infarction (16%). In rare cases, HOD can occur without injury to the DROP and can be accompanied by progressive ataxia and palatal tremor, so-called PAPT-syndrome, which is idiopathic and typically a bilateral process [10,11,19,20,21]. The development of HOD after focal radiotherapy such as Gamma-Knife with injury to the DROP was described in a case series by Yun et al. for four patients and in a single case report of radiosurgery for pontine metastasis, but did not occur in our series [22,23].

Furthermore, we demonstrated that despite the presence of tumors in the posterior fossa, including mass effects and—to a certain degree—tumor infiltration, HOD was not present in all patients’ pre-therapeutic MRI examinations, suggesting that tumor was not the cause of HOD. However, post-therapeutic MRIs revealed HOD within the expected time window in all patients [7]. Thus, in this selected collective of neuro-oncological patients, analysis revealed that surgical lesions in the dentate nucleus with hemorrhagic tissue damage were the most likely cause of HOD. Hence, we hypothesize that it is more often the interventions themselves and not the tumor that cause HOD. 

Even though the available data are highly suggestive, the development of HOD in the cases presented had there not been an operative intervention cannot be ruled out entirely. However, none of the patients in this study showed any signs of HOD on MRI acquired within 3 months prior to surgery. In a recent case series, Hirano et al. suggested that HOD develops either primarily as a result of the tumor mass itself, or secondarily as a result of treatment [15]. Hirano et al. also included patients with posterior fossa masses of non-tumor origin. In contrast to our findings, 3 out of 10 patients had already developed HOD before or without neurosurgery. In our cohort, all patients with visible lesions developed HOD only after therapeutic intervention. The discrepancy of the results may be explained by small sample sizes and by the different (non-tumor) etiologies of the cases reported by Hirano and colleagues.

Naturally, the likelihood of HOD development in our patients in the absence of therapeutic interventions remains speculative. Nevertheless, in both cohorts, the majority of patients showed signs of HOD only after therapeutic interventions, including neurosurgery. Both analyses share the limitation of retrospective design and relatively small cohort size with heterogeneous diagnoses and differing therapeutic regimens. Prospective studies are warranted in order to verify the causality between HOD and therapeutic interventions. 

To date, therapeutic options for HOD symptoms have limited evidence and are strictly symptomatic but non-causal. According to literature, palatal tremor as well as Holmes tremor can be treated with either high-dose dopamine, anticholinergics, clonazepam, clozapine, levetiracetam, or high-dose trihexyphenidyl [24,25,26]. Stereotactic treatments can be considered in drug-resistant cases and relevant disability [26,27]. Deep brain stimulation of the red nucleus on both sides was proven to be unhelpful in a case with (oculo-)palatal tremor [28].

The lack of causal medical regimens to treat HOD and its symptoms stresses the need for improved prophylactic measures against HOD occurrence. In perspective, medical and interventional measures to prevent the development of HOD, as well as therapeutic approaches to ameliorate HOD symptoms should be evaluated.

Future studies should choose a multicenter prospective design to determine the incidence of HOD in neuro-oncological patients with posterior fossa masses as well as the frequencies of HOD following different neurosurgical approaches. 

## 5. Conclusions

We demonstrated that strategic lesions in the posterior fossa within the Guillain–Mollaret triangle may lead to unilateral or bilateral HOD. Destructive lesions of the dentate nuclei after therapeutic measures, including neurosurgery, were associated with occurrence of HOD. In this selected cohort of 12 patients with posterior fossa masses, HOD was not present before the therapeutic intervention for the tumors took place. Even though gradual onset of novel symptoms over months is not uncommon in neuro-oncological patients, the presence of palatal tremor, Holmes tremor, or pendular nystagmus is highly suggestive for HOD. Diagnosing and recognizing HOD requires awareness of the pathophysiology, imaging findings, and clinical presentation.

## Figures and Tables

**Table 1:** Neuro-oncological patient characteristics.

Case	Age, Sex	Etiology	Tumor Localization	Surgery	Surgical Approach (side)	Non-Surgical Therapy
1	15, male	Medulloblastoma	Fourth ventricle, Lepto-meningeal and spinal	Partial resection	Transvermal (midline)	RCH (craniospinal RT (41 Gy), posterior fossa + tuber cinereum boost (14.4 Gy), MET-HIT 2000-AB4-M2-4)
2	60, male	Brain metastasis (adenocarcinoma)	Cerebellum, right	Resection	Paramedian (right)	-
3	26, male	Medulloblastoma	Fourth ventricle	Resection	No data	RCH (craniospinal RT (40 Gy), posterior fossa boost (60 Gy), residual tumor boost (68–72 Gy), HIT-SKK)
4	56, male	Glioblastoma	Mesencephalon	Biopsy	Frontal biopsy (right)	RCH (RT (46 Gy), boost (8 Gy), temozolomide)
5	19, female	Medulloblastoma	Fourth ventricle	Resection	Telovelar (midline)	RCH (craniospinal RT (23.4 Gy), posterior fossa boost (19.8 Gy), vincristine)
6	71, female	Glioblastoma	Temporopolar, right	Resection	Temporal (right)	RCH (60 Gy, temozolomide), CH (dose-dense temozolomide), Re-RT (20 Gy)
7	35, male	Medulloblastoma	Fourth ventricle	Resection	Telovelar, transvermal (midline)	RCH (craniospinal RT (35.2 Gy), posterior fossa boost (19.8 Gy), vincristine, lomustine, cisplastin)
8	56, female	PCNSL	Pons, thalamus, and centrum semiovale, right	Biopsy	Parietal biopsy (right)	CH (rituximab, methotrexate, Ara-C)
9	28, male	Papillary tumor of the pineal region (WHO II)	Fourth ventricle	Resection	Telovelar (midline)	Brachytherapy, radiosurgery (15 Gy)
10	63, male	Cerebellar metastasis (SCLC)	Cerebellar vermis	-	No surgery	RCH (WBRT (30 Gy), mediastinal RT (50 Gy), cisplatin, etoposide)
11	41, male	Medulloblastoma	Fourth ventricle, left dentate nucleus	Partial resection	Telovelar (midline)	RCH (craniospinal RT (35.2 Gy), posterior fossa boost (19.8 Gy), vincristine)
12	12, female	Ependymoma (WHO II)	4th ventricle	Biopsy, resection	Transvermal (midline)	CH (methotrexate, etoposide, cyclophosphamide, vincristine, carboplatin, Ara-C), RT (craniospinal RT (36 Gy), tumor boost (20 Gy))

Neuro-oncological patient characteristics are shown. Age is defined as age at the time of the initial diagnosis of the neuro-oncological disease. Treatments are only described when conducted before the occurrence of HOD. The surgical approaches are listed as taken from the OR report. Abbreviations: SCLC = small cell lung cancer, RCH = radiochemotherapy, (WB)RT = (whole brain) radiation therapy, CH = chemotherapy.

**Table 2:** Hypertrophic olivary degeneration (HOD) characteristics.

Case	Age, Sex	Diagnosis	HOD on Preoperative MRI (latency) ^1^	HOD on First Postoperative MRI (latency) ^2^	Latency Surgery to HOD (months)	Lesion Localization within Dentato-Rubro-Olivary Pathway	HOD Side	HOD Symptoms (PT, HT, PN)	HOD Dx
1	15, male	Medulloblastoma	no (−1d)	no (+8d)	4	bilateral dentate nucleus	right	no	n. a.
2	60, male	Brain metastasis (adenocarcinoma)	no (−8d)	yes (+4w)	1	right dentate nucleus	left	PT	yes
3	26, male	Medulloblastoma	n. a.*	no (+6w)	9	bilateral dentate nucleus	bilateral	n. a.	no
4	56, male	Glioblastoma	no (−1d)	no (+4m)	7	left sup. cerebellar peduncle	right	PT	yes
5	19, female	Medulloblastoma	no (−6d)	no (+1d)	8	bilateral dentate nucleus	bilateral	PT	no
6	71, female	Glioblastoma	no (−4d)	no (+3d)	30	right sup. cerebellar peduncle	left	no	no
7	35, male	Medulloblastoma	no (−3d)	no (+2d)	10	bilateral dentate nucleus	bilateral	no	no
8	56, female	PCNSL	no (−2d)	no (+1d)	1	right red nucleus and central tegmental tract	right	no	no
9	28, male	Papillary tumor of the pineal region (WHO II)	no (−13d)	no (+1d)	2	right dentate nucleus	left	no	no
10	63, male	Cerebellar metastasis (SCLC)	no surgery	no surgery	no surgery	no visible lesion	right	no	yes
11	41, male	Medulloblastoma	no (−31d)	no (+2d)	3	left dentate nucleus	right	HT	yes
12	12, female	Ependymoma (WHO II)	n. a.*	no (+1d)	4	bilateral dentate nucleus	left	n. a.	no

HOD characteristics are shown. Age is defined as age at the time of the initial diagnosis of the brain tumor. ^1^ Time latency between last MRI without signs of HOD and neurosurgery is provided in days, weeks, or months, e.g., (−2d) indicates MRI without signs of HOD was acquired 2 days before neurosurgery. ^2^ Time latency between surgery and first postoperative MRI without signs of HOD, provided in days, weeks, or months, e.g., (+2d) indicates that postoperative MRI without signs of HOD was acquired 2 days after neurosurgery. Latency to HOD refers to the time interval between neurosurgical intervention and first signs of HOD on MRI in retrospective image analysis. HOD diagnosis refers to the mentioning of HOD within the discharge letter by the treating physician. * Patients were referred to our center for post-operative therapy after external neurosurgery; therefore, pre-operative imaging (MRI) was not available retrospectively.
